# Experimental Assessment of Sleep-Related Parameters by Passive Infrared Sensors: Measurement Setup, Feature Extraction, and Uncertainty Analysis

**DOI:** 10.3390/s19173773

**Published:** 2019-08-31

**Authors:** Sara Casaccia, Eleonora Braccili, Lorenzo Scalise, Gian Marco Revel

**Affiliations:** Department of Industrial Engineering and Mathematical Sciences, Polytechnic University of Marche, 60131 Ancona, Italy

**Keywords:** PIR sensor, home measurements, sleep-related parameters, sleep latency, sleep interruptions, time to wake, sleep efficiency

## Abstract

A simple sleep monitoring measurement method is presented in this paper, based on a simple, non-invasive motion sensor, the Passive InfraRed (PIR) motion sensor. The easy measurement set-up proposed is presented and its performances are compared with the ones provided by a commercial, ballistocardiographic bed sensor, used as reference tool. Testing was conducted on 25 nocturnal acquisitions with a voluntary, healthy subject, using the PIR-based proposed method and the reference sensor, simultaneously. A dedicated algorithm was developed to correlate the bed sensor outputs with the PIR signal to extract sleep-related features: sleep latency (SL), sleep interruptions (INT), and time to wake (TTW). Such sleep parameters were automatically identified by the algorithm, and then correlated to the ones computed by the reference bed sensor. The identification of these sleep parameters allowed the computation of an important, global sleep quality parameter: the sleep efficiency (SE). It was calculated for each nocturnal acquisition and then correlated to the SE values provided by the reference sensor. Results show the correlation between the SE values monitored with the PIR and the bed sensor with a robust statistic confidence of 4.7% for the measurement of SE (coverage parameter k = 2), indicating the validity of the proposed, unobstructive approach, based on a simple, small, and low-cost sensor, for the assessment of important sleep-related parameters.

## 1. Introduction

Sleep conditions, in terms of duration and interruption, change as a function of the normal aging process [1]. However, those that initially may seem to be aging-related changes, can conceal non-negligible sleep disorder symptoms. Sleep disturbances and cognitive impairment are common in older adults and findings from observational studies provide evidence that some sleep disturbances (i.e., sleep duration, sleep fragmentation, and sleep-disordered breathing) are related to the development of cognitive impairment [1]. Sleep disorder pathologies are associated with reduced quality of life, increased morbidity, and higher mortality risk, thus representing a significant economic and social burden to society [2]. In patients affected by dementia, an altered sleep pattern (as described in Section 1.1) is frequently reported and possibly justified by the possible irreversible damage to the brain areas responsible for sleep regulation reported for some dementing illness, such as Alzheimer’s disease, Parkinson’s disease, etc. [3]. 

It is therefore imperative to diagnose sleep disorders early and accurately [3] in order to detect the initial symptoms of the degenerating pathologies early and to slow down their effect with appropriate actions. Till today, a great number of specialized sensing systems have been designed for sleep monitoring, but they often result to be intrusive, uncomfortable, obtrusive, and/or expensive (as reported in Section 1.2) and consequently of difficult use in practical cases. The challenge is to find a solution that may overcome these drawbacks and be efficient as well.

At present, PIR sensors are commonly used to monitor the behavior of users inside the home environment, measuring activities of daily living (ADLs). For example, the use of PIR sensors installed in the bedroom, bathroom, etc., allows to assess the ADLs of the users and to tailor specific services for them [4,5,6]. This paper is focused on the proposal of a measurement set-up based on the use of a simple, cheap, non-invasive, and non-contact Passive InfraRed (PIR) motion sensor for the assessment of sleep parameters. To this purpose, this work proposes a specific experimental set-up for monitoring sleep and individuates a set of parameters for the evaluation of the sleep states using a single PIR sensor. The measured parameters were correlated with the sleep features extracted from a bed sensor to validate the uncertainty in the measurement of such parameters [7]. The use of PIR sensors keep the cost low and the size of produced data-files small, with the purpose to increase easiness of use and uptake. Moreover, the PIR sensor is perfectly fitted for sleep monitoring of a user at home, in hospital, or in a nursing home, in respect to the important privacy issue aspects [8]. The correlation between the PIR sensor and the reference ballistocardiographic bed sensor, in the monitoring of sleep phases, was computed aware that bed sensors have less accuracy in relation to the gold standard techniques, as reported in the literature [9,10].

### 1.1. Sleep Stages

In this paragraph, the sleep patterns and features used for this work are described. There are two basic phases of sleep rapid eye movement (REM) sleep and non-REM (NREM) sleep. Typically, REM sleep first occurs about 90 min after falling asleep. During this sleep state, eyes move rapidly from side to side behind closed eyelids. Mixed frequency brain wave activity becomes closer to that seen in wakefulness. Breathing becomes faster and irregular and heart rate and blood pressure increase to near waking levels. Normal adults spend about 20% of total sleep time in REM [11] states. NREM sleep is characterized by unconsciousness and increased parasympathetic tone, which is expressed by a fall in blood pressure and slowing in respiration. Four phases are considered for clinical purpose. In Stage 1 (Falling asleep) the brain waves begin to slow from their daytime wakefulness patterns. At this stage, the person can be easily awakened but may have the feeling of not having rested enough or not having slept. In Stage 2 (Light sleep) the heart-beat and breathing decrease, muscles relax even further, body temperature drops, and eye movements stop. Stages 3 (deep sleep) and 4 (very deep sleep) are considered as Deep sleep and starts 35–45 min after falling asleep. Heartbeat and breathing slow to their lowest levels, muscles are relaxed, and it may be difficult to awake. Normal adults spend 25% of total sleep in Stage 3 [11]. Sleep is characterized by multiple dimensions, each of which is important to assess a qualitative and quantitative analysis of the sleep pattern [12]. The sleep patterns considered for this work are the Sleep Duration, Sleep Efficiency, Awakenings, Sleep Latency, and Time to Wake. The Sleep Duration or Total Sleep Time (TST) is the amount of sleep time considering the total of all REM and NREM sleep. The Sleep efficiency (SE) is the proportion of sleep in the episode potentially filled by sleep. It is the ratio of TST and Time in Bed (the time from “light off” to “light on”). The sleep interruptions (INT) are the awaked states from any NREM to REM sleep stages. Generally, the interruptions which have a certain duration (equal or more than 5 min) are classified as awakenings [13]. Important parameters for the assessment of the sleep stages are Sleep Latency (SL), which is the amount of time that the user takes to fall asleep from “light out” bedtime, and Time to Wake (TTW), which is the time that the person spends staying in bed after waking. 

### 1.2. Sleep Monitoring Sensors

Monitoring sleep parameters depends on the chosen sensors. Several kinds of sensors and devices are in the market to measure sleep activity. From a clinical and research standpoint, the capacity to obtain longitudinal sleep-wake data may improve disease phenotyping, individualized treatment decisions, and health optimization. From a wellness standpoint, commercially available devices may allow individuals to track their own sleep with the goal of finding patterns and correlations with modifiable behaviors such as exercise, diet, and sleep aids [14]. 

Polysomnography (PSG) is considered the gold standard measurement system to assess sleep physiology in health disease. PSG measures many body functions to identify sleep disorders, including brain activity (electroencephalography—EEG), eye movements (electrooculography—EOG), muscle activity (electromyography—EMG), and heart rhythms (electrocardiography—ECG) during sleep. This system is largely used in clinical practice while portable sensors have been developed for the evaluation of sleep at home. Brain-activity sensors are the sensors category closer to PSG. iBrain and Zeo are examples of commercially available portable devices used to measure sleep features. iBrain measures a single frontal EEG signal and Zeo monitors a combination of EEG, frontalis muscle EMG, and EOG [14]. Wearable sensors are also used in sleep-related studies. They are worn by the user to monitor body activity (e.g., accelerometers, gyroscopes, and magnetometers) and physiological parameters (e.g., electrocardiogram signals, heart rate, breathing rate, skin temperature). Several wearable sensors to monitor sleep patterns are available on the market. The wearable sensors to monitor the sleep activity are based on the measurement of physiological parameters, e.g., heart rate and breathing rate, and activity and body movements. Fitbit, Lark, Sleep Cycle Alarm, Sleep tracker, and Wake Mate are wrist-bands to monitor sleep features. BioHarness, HealthVest, and LifeBed are some examples of devices not directly measuring sleep features, but only physiological parameters and activity information [14]. A growing demand for inexpensive systems to monitor older people, and also infants, at their homes has motivated the development of bed sensors. A bed sensor is a special mattress or sensor based on ballistocardiography [15] placed under the mattress. Bed sensors can extract bed occupancy information including time in bed, number of bed exits, and of first morning exit, even the heart rate of the user. Air Cushion, EarlySense Mattress, and Emfit Bed Sensor are examples of bed sensors to monitor sleep patterns [14].

Recent studies have tested a cost-effective and unobtrusive way to remotely monitor changing sleep pattern over time by using Passive InfraRed (PIR) motion sensors. A multiple PIR sensor network distributed in the home can derive sleep parameters such as bed time, rise time, sleep latency and nap time if associated with specific algorithms based on different rules [16,17].

The development of home assistance devices is moving toward the realization of products that are unobtrusive, non-intrusive, simple to use, and accurate as well. The cost of devices is another fundamental aspect to be considered. Bed sensors have a basic cost in the order of hundreds of euros that rises in respect to the outputs provided by the sensor itself. Wearable sensors have a variable cost respect to the chosen sensor, but in the Active and Assisted Living (AAL) field using non-contact sensors is preferred to avoid change in the user attitude caused by the application of a wearable sensor [18]. PIR sensors are in the low-cost range (about 10–20 euros) among the sensors previously described. PIR sensors are unobtrusive, non-intrusive, and easy to use and install. However, till today there are only few studies about its accuracy in the assessment of sleep [16,17]. 

## 2. Materials and Methods

The present work is based on the realization of a measurement set-up based on the use of a PIR sensor for the measurement of sleep patterns. Twenty-five nights of nocturnal data acquisition, for a healthy user, were included in the analysis and signals processing, and analysis of data and algorithm development were done to complete the study. 

### 2.1. Measurement Set-Up

**Figure 1:**
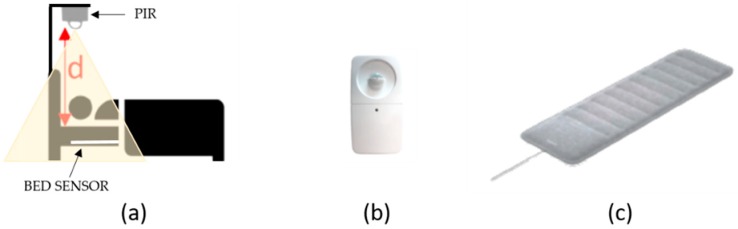
(**a**) The measurement set-up during the test nights with the user lying in bed and the Passive InfraRed PIR sensor installed at a distance d of 1.10 m above the pillow; (**b**) PIR sensor; (**c**) reference bed sensor (Nokia sleep bed sensor) installed under the mattress.

The measurement set-up was characterized by the installation of a PIR sensor (Figure 1b) at a distance of 1.10 m (Figure 1a) above the pillow using a support and the placement of a ballistocardiographic bed sensor (Nokia Sleep bed sensor) under the mattress (Figure 1c). 

A PIR sensor is a pyroelectric device that senses InfraRed (IR) radiation changes within its viewing range. The IR energy of an object depends on different parameters such as temperature, color, and texture. Ordinary PIR gives a logical one or a logical zero as its output, depending on whether the movement is detected or not [19]. The PIR sensor considered for this work, Figure 1b, has the following technical characteristics:

- Operative range: 5 m;
- Field of view: 140° (horizontal);
- Time delay: 2 s;
- Measurement range: −20–40 °C;
- Trigger mode: Continuous sensing;
- Working Voltage: 3 V.

The bed sensor was used as a reference sensor [20] to validate the PIR sensor in the measurement of the sleep phases. The bed sensor was used as a reference sensor in this work because these sensors are frequently installed in the home environments of AAL frameworks. The bed sensor is based on the ballistocardiographic technology. In the literature, ballistocardiographic sensors are compared with the gold standard PSG in the measurement of REM, non-REM, and AWAKE phases with a total accuracy of 76.81% ± 7.51% using a time-variant autoregressive model and quadratic classifier to extract features [9] and movements with 95% and 96% probability of detection and 94% and 95.2% accuracy in sleep and restlessness state identification, respectively [10].

The bed sensor used for this work is a rectangular pad (Figure 1c) that fits under the mattress, at the chest level (dimensions: 64 × 19 cm, thickness: 5 mm, weight: 350 g). The sleep sensor is made of a sensitive pressure sensor that utilizes the principle of ballistocardiography to measure the presence and (respiration rate and heart rate) of the user sleeping. It mainly provides an accurate distinction of the sleep cycle into the phases of main interest: AWAKE (SL, TTW, and INT), REM, DEEP, and LIGHT. Furthermore, the bed sensor can measure sleep duration and track snoring. All the mentioned sleep parameters are then combined to give an overall score of the night, ranked on a scale of 0–100, indicating the quality of the sleep. The bed sensor was synchronized automatically with the Nokia Health Mate app to collect all the data, the app providing data history, personalized advice, and coaching programs, including one developed specifically for sleep. 

#### Preliminary Investigation

A preliminary study was conducted on four healthy users (two men and two women) to evaluate the accuracy of the PIR sensor in measuring the movement of a lying user in bed. The user was covered up to the neck, and the only IR source was represented by the head: the blanket acts as a shield for the underlying body heat, hence any movement occurring under it is not detectable (including breathing). This choice was made to test the PIR sensor in the most critical condition. In this way, the IR emission surface was reduced to the minimum. Each user was asked to move the head from side to side starting from the frontal position. The movements were done with no displacement on the pillow, just a rotation of the head on the transversal plane. The number of movements detected by each sensor was identified in post-processing using Matlab. A total of 100 head movements were performed at a distance of 1.10 m. The installation of the PIR sensor above the pillow shows a movement accuracy detection of 85% (on average), in accordance with the technical characteristics of the used PIR sensor and the results shown in [21,22]. 

### 2.2. Data Acquisition

Twenty-five nights of nocturnal data acquisition were performed by using simultaneously the bed and PIR sensor on one voluntary, healthy user. 

The subject was covered up with a blanket up to the neck and the only thermal radiation detectable by the PIR sensor was represented by the user’s head. 

#### 2.2.1. Data Analysis and Processing

**Figure 2:**
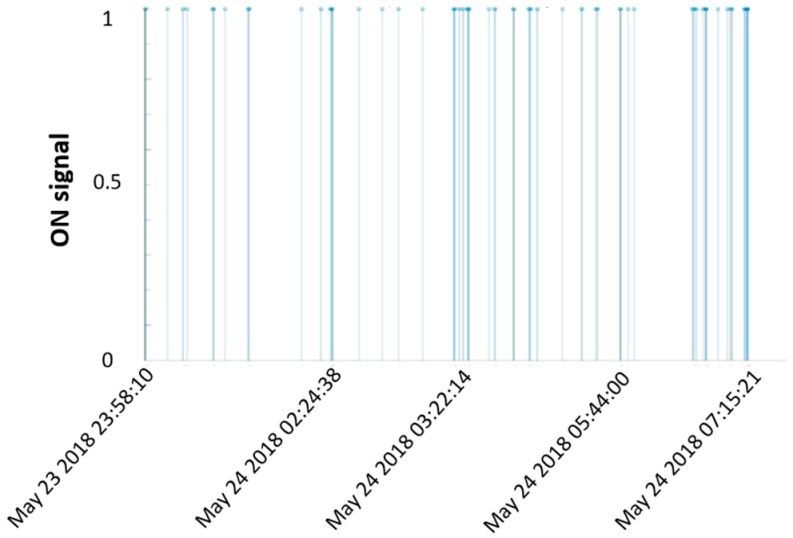
PIR sensor: one night example of data collection; the PIR sensor “ON” is displayed. On the x-axis, the timestamps refer to each night movement detection (not all the PIR date events are displayed to make the figure readable).

The PIR data were collected in ASCII file as a series of movements detection/sensor inactivation logs. The file includes all the logs created during the whole acquisition period. Since the sensor was fixed at the validated location and kept connected continuously for one month, also occurrences outside the sleep episodes were included in the logs. For this reason, the files were cut out so as to exclude any external event: each night was identified referring to the start/end timestamp provided by the Nokia Health Mate® app, that gives (also) the information about the exact moments in which the user lies down/gets up. PIR sensor signals were then pre-processed in order to be compared to the bed sensor outputs: the raw data were converted in to a series of logical 1 and 0 for each movement detection (ON)/sensor inactivation (OFF), respectively. For each “ON”, timestamp was also reported (Figure 2).

#### 2.2.2. Algorithm

**Figure 3:**
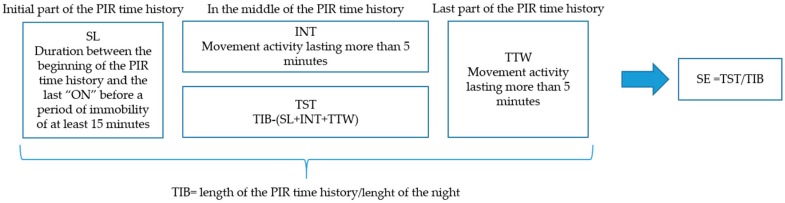
Sleep patterns extracted from the algorithm and the related method used to derive these parameters.

A specific algorithm was developed to extract some sleep patterns from the PIR signals. The algorithm was focused on the time in which the activation and deactivation of the PIR sensor occur. The SL, TTW, INT, and TIB phases were measured considering the time of the PIR occurrences, and TST and SE were derived from the previous parameters. By the observation of the data and according to previous work [23], it was possible to rely on a period of immobility to characterize the definition of the SL period. SL is always in the first part of the PIR time history and a minimum of 15 min of immobility was observed to define the transition from the wake state to the sleep state. SL was defined as the duration between the beginning of the signal and the last “ON” before a period of immobility of at least 15 min. INT episodes and the TTW were instead identified as phases characterized by a certain number of movements (a train of “ON”) that occur within 6 min. INT was identified in the middle of the PIR time history, while TTW was always at the end of the PIR time history. Trains of movements (ON) that lasted less that 5 min were not considered as sleep interruptions because the sleep interruptions need more than 5 min to be detected [13]. Hence, TST (total sleep time) was calculated by subtracting the summation of the waking periods from the duration of the whole night; TIB was instead measured as the duration of the whole night (start/end of the acquisition). As a consequence, sleep efficiency was computed. Sleep efficiency was calculated also with the measurements provided by the Nokia app. Figure 3 summarizes the sleep patterns extracted from the algorithm and the related method or formula used to derive these parameters.

#### 2.2.3. Feature Extraction

The bed sensor shows a complete distinction of the sleep cycle into the phases of main interest: AWAKE, REM, DEEP, and LIGHT [24]. 

**Figure 4:**
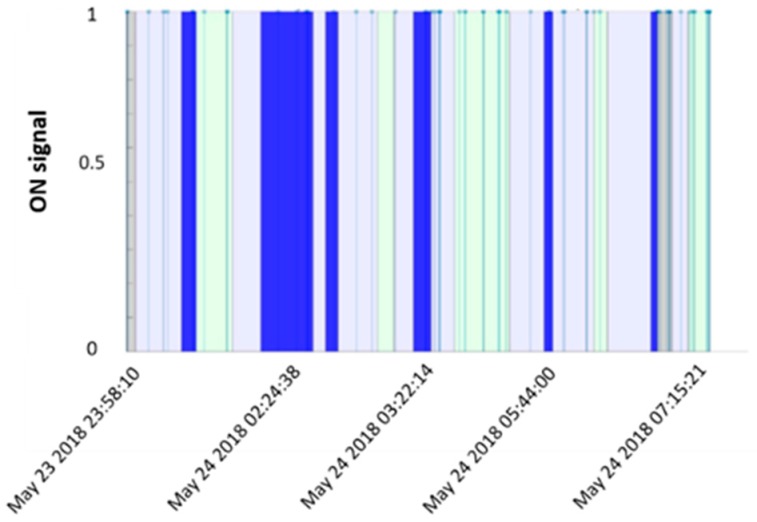
Sleep pattern from PIR and reference sensor output. In grey, AWAKE phases; in dark blue, DEEP phases; in light green, rapid eye movement (REM) phases; in light blue, LIGHT phases (not all the PIR date events are displayed to make the figure readable).

Since the aim of this research is to check the possibility to extract features from the PIR signal for the recognition of sleep phases, the two sensors output were overlapped (Figure 4). 

Start/end phase timestamps of the reference sensor (from the app) were uploaded to the pre-processed PIR sensor signal. The only information that may be deduced from the PIR sensor signal is the number of movements that occurred, thus their distribution all over the night. By combining this few information with the phases start/end provided by the reference sleep sensor, a “body movement index” (BMI) was computed. BMI represents the occurrence of body movements in each sleep stage, thus it is defined by the ratio:

(1) $$\mathrm{BMI}=\;\frac{\mathrm{Number}\;\mathrm{of}\;\mathrm{body}\;\mathrm{movements}}{\mathrm{Duration}\;\mathrm{of}\;\mathrm{sleep}\;\mathrm{phase}\;\left(s\right)}\times \;100.$$

In this way, each phase (AWAKE, REM, DEEP, LIGHT) was associated to a BMI. This computation was repeated for each phase episode of the night, for all the acquired nights. In addition, the sleep efficiency (SE) was measured. SE is defined as the ratio in Equation (2):

(2) $$\mathrm{SE}=\;\frac{\mathrm{TST}}{\mathrm{TIB}},$$

where TST = time spent asleep (Deep, Light, REM phases), and TIB = TST + Time spent awake (SL + INT + TTW).

#### 2.2.4. Statistical Analysis 

**Figure 5:**
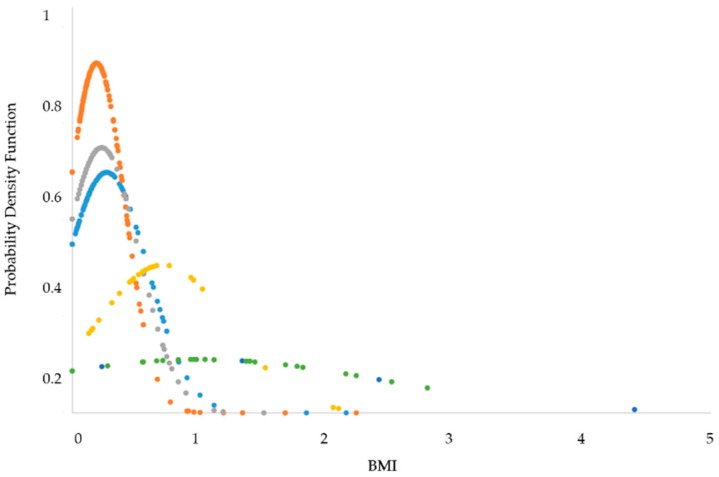
Body movement index (BMI) data distribution. In orange, class LIGHT; in grey, class REM; in dark blue, class DEEP; the class AWAKE: in yellow, Sleep Latency (SL); in green, Sleep Interruptions (INT); in light blue, Time to Wake (TTW).

Average (AVG) and standard deviation (SD) of the BMIs of the same phase class were calculated. Then, the probability density function (PDF) was computed by using the AVG and SD previously evaluated (Figure 5). 

In this way, it was possible to graph the BMI distributions of each phase and evaluate if the four classes (REM, DEEP; LIGHT, and AWAKE) are superimposed on each other (they are not distinguishable) or have distributions different enough to be recognized among all the phases. 

Wilcoxon test was done by using MATLAB. This test gives a measure about the meaningfulness of the BMI difference among the classes. The Wilcoxon test determines if the BMI values computed for each class are characteristic of that class or are too similar to each other not to allow a distinctive characterization of the class. To test the meaningfulness of the data, alpha-value was set at 0.05 (significance level). The *p* value is the statistical value that characterizes if the hypothesis is statistically significant or not. For this work, if *p* > 0.05 the null hypothesis is 0, otherwise the null hypothesis is 1. When the null hypothesis (*p* < 0.05) is 1, the correlated classes are statistically significant [25]. In addition, since the duration of each phase is variable during the night (i.e., REM sleep episodes become longer in respect to the first part of the night, typically reaching their longest duration in early morning), it was also analyzed whether the number of body movements in the sleep phase are related to the duration of the phase. To this purpose, the R value and R-squared (R^2^) value were computed. The R value or Pearson correlation coefficient is a measure of the linear correlation between two variables. R^2^ is a statistical measure of how close the data are to the fitted regression line [26].

#### 2.2.5. Uncertainty Analysis 

An uncertainty analysis was undertaken on the sleep efficiency. 

To evaluate how accurate SL, INT, TTW and SE measurements were, relative mean difference and uncertainty were computed.

Then, to test the algorithm reliability in the detection of the AWAKE phases, the Precision Algorithm (Equation (3)), which represents the fraction of how many positively classified episodes are true positives, was computed.

(3) $$Precision=\frac{True\;Positive}{True\;Positive-False\;Positive}.$$

## 3. Results

Twenty-five nights corresponding to 200 h of nocturnal acquisitions (including 162 DEEP, 246 LIGHT, 111 REM, 20 INT phase events, identified by the reference bed sensor), were acquired by simultaneously using the PIR sensor (above the head of the subject at 1.10 m of distance) and the bed sensor (the reference tool in this study) under the mattress.

### 3.1. Statistical Analysis

In the following table (Table 1) the R-values and R^2^ values computed for each phase are shown. Correlation (R-value) between phase duration and body movements number occurred in the same phase was calculated for DEEP, LIGHT, REM, and AWAKE phases to understand if the quantity of movements in each of then varies respect to the duration. Additionally, R^2^ values were computed to test if the movement occurrences were random events or not. The study of these values is critical to understand if, for the phase recognition, it is possible to rely on the number of movements that occurred in a certain time duration or not.

These results promote the possibility to further the study, mainly for the recognition of the waking state. In particular for REM and DEEP phases, the occurrence of movements with respect to the phase duration was completely random (DEEP: R^2^ = 0.0009; REM: R^2^ = 0.1662). No correlation was found between these two variables for these phases (R < 0.40). Higher values of both correlation coefficient R, and coefficient of determination R^2^ were instead obtained for the other phases: LIGHT and AWAKE (SL, INT, and TTW). This statistical analysis confirms that, in particular during the waking phases, there is more movement throughout the duration of the phase: they are not sporadic but significant movements of lasting “agitation” state. The high R^2^ value also confirms the non-randomness of the movements (data is not widespread). Already from this first statistical analysis, the distribution of the movements in each phase is particularly different between the waking and sleep class of phases. These results are motivated from the measured signal of the PIR sensor. The PIR sensor measures a series of 1 and 0, corresponding to the movement of the user. The sensors to monitor the REM and DEEP phases use more parameters than just the movement, e.g., heart rate, breathing rate, etc. [11,14]. Therefore, the PIR sensor can be used to monitor the sleep phases that are not just focused on physiological parameters but for the most part on movements. 

The statistical results were translated in terms of BMI, characteristic of each phase, and BMI index was computed. The averaged BMI and SD values of each phase are reported in Table 2.

Each phase is represented by a different BMI value. This may suggest the possibility to process the PIR sensor signals by referring to the respective BMI value to discriminate each phase. However, the high variability of these BMI values, as confirmed by the high SD, can cause many false positives, due to the overlapping of the phase classes. For this reason, a Wilcoxon test was performed (Table 3) to define if there were meaningful differences among the BMI values or if these values were too close to not allow phase recognition.

Wilcoxon test results on BMIs confirmed meaningful differences between awake/asleep classes of phases. No significative differences resulted among the phases belonging to the same class (*p* > 0.05), except for the comparison of SL-TTW and DEEP-LIGHT.

### 3.2. Uncertainty Analysis

The results above allowed the development of the algorithm for the recognition of the AWAKE phase, specifically focusing on the identification of the SL, INT, and TTW which are the useful information for a sleep pattern evaluation. 

The Precision Algorithm was computed for each AWAKE class. True positive and false positive events were considered for this calculation. 

SL and TTW events were identified by the algorithm with a precision of 100%. Instead, interruption events detected by the algorithm exceed the ones reported by the reference sensor: a high number of false positive interruption events were identified by the algorithm, leading the algorithm precision to the low value of 17% (Table 4). This can be explained by the fact that the light phase, by definition, is a phase of transition from the state of wake to deeper sleep phases, and it is also characterized by body movements, as evident in Table 1. In this case, the algorithm was not able to distinguish the wake state from the light phase and many movements that occurred in such phase were denoted as interruptions.

The AWAKE phases (SL, INT, and TTW respectively) measured by the developed algorithm were compared with the ones computed by the reference tool.

The values of statistical confidence, with a coverage factor equal to two, are reported in Table 5.

**Figure 6:**
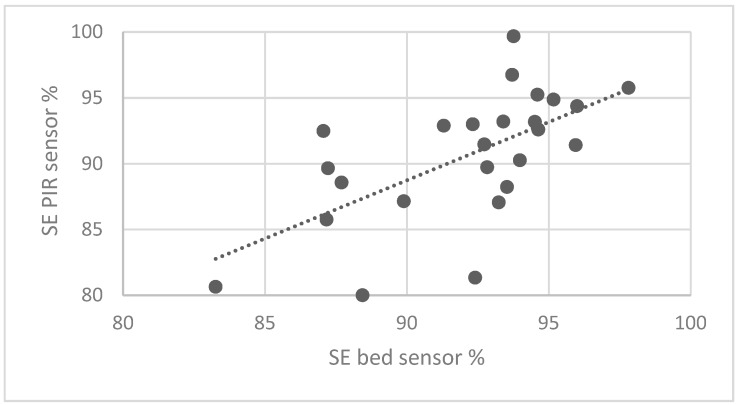
Plot and linear regression for the sleep efficiency (SE) parameters.

SE values were then computed for each night, by using the events detected by the algorithm. The SE values computed by the algorithm are very close to the ones computed with the bed sensor measurements (pre-processed). Figure 6 shows the plot and the linear regression related to SE.

## 4. Discussion

This paper focused on the validation of a PIR sensor to monitor sleep patterns correlating the PIR sensor and the bed sensor in the monitoring of sleep parameters. The use of PIR sensors in smart homes provides benefits regarding privacy and security for the users. Considering older people, the benefits increase because the PIR sensors do not need active intervention from the users [27]. The PIR sensor networks are used in the AAL field [28] to monitor the Activities of Daily Living of older people [29], to improve their life quality [30] and to make them independent [25]. The cost of a single PIR sensor is one order lower in respect to the cost of a home sensor network to monitor behavior of the user or a bed sensor. For these reasons, this work focused on the use of a single PIR sensor to monitor the sleep patterns that are important indicators of aging pathologies, e.g., dementia and diseases, e.g., depression [31,32]. Twenty-five nights of measurements were acquired on a healthy user and the data acquired from the PIR sensor and the bed sensor were correlated to define the capability of the PIR sensor in the detection of sleep characteristics. Results showed that with the PIR sensor it is possible to detect the waking state, better than REM, DEEP, and LIGHT, i.e., R < 0.61 and R^2^ < 0.37. This result is based on the fact that to measure such complex sleep phases it is necessary to also monitor physiological parameters, e.g., heart rate, breathing rate, etc. A high correlation is visible between the BMI that occurred and the AWAKE (SL, INT, TTW). To improve this result, the Wilcoxon test was used, and results confirmed meaningful differences between awake/asleep classes of phases. The algorithm developed attempted to identify, with good accuracy, the SL parameters considering the waking state parameters but the Sleep Efficiency (SE) computation shows the best results. The SE values measured from the PIR data are very close to the ones computed with the reference bed sensor measurements (statistic confidence, with a coverage factor equal to two: 4.7%) and this is an important aspect considering that the SE parameter is used to estimate the mortality risk in elders [33].

## Figures and Tables

**Table 1:** R and R^2^ values computed for each phase: DEEP, LIGHT, REM, and AWAKE (SL, INT, and TTW).

Sleep Phase	R Value (Pearson)	R^2^ Value
DEEP	−0.03	0.00
LIGHT	0.61	0.37
REM	0.40	0.16
SL	0.67	0.45
INT	0.71	0.55
TTW	0.86	0.69

**Table 2:** Average (AVG) and standard deviation (SD) of the BMI obtained for each phase.

Sleep Phase	BMI (AVG)	BMI (SD)
DEEP	0.27	0.32
LIGHT	0.19	0.27
REM	0.23	0.29
SL	0.71	0.52
INT	1	1.44
TTW	1.14	0.73

**Table 3:** Statistical results from the Wilcoxon test.

Sleep Phases	Null Hypothesis	*p*-Value
DEEP-LIGHT	1	0.001
DEEP-REM	0	0.175
DEEP-SL	1	0.000
DEEP-INT	1	0.037
DEEP-TTW	1	0.000
LIGHT-REM	0	0.223
LIGHT-SL	1	0.000
LIGHT-INT	1	0.008
LIGHT-TTW	1	0.000
REM-SL	1	0.000
REM-INT	1	0.023
REM-TTW	1	0.000
SL-INT	0	0.296
SL-TTW	1	0.008
INT-TTW	0	0.676

**Table 4:** The Precision Algorithm and detection accuracy of the AWAKE phases (SL, INT, and TTW) event detection.

Sleep Phase	Precision (%)
SL algorithm detection	100
INT algorithm detection	17
TTW algorithm detection	100

**Table 5:** Statistical confidence, with a coverage factor of two, correlating the PIR sensor measures with the reference sensor.

Sleep Phase	Uncertainty (SD)
SL	23.9%
INT	56.1%
TTW	49.5%
